# Essentials of Minor Surgery

**Published:** 1893-12

**Authors:** 


### BOOK NOTICES.

ESSENTIALS OF MINOR SURGERY. Bandaging and Venereal Diseases, arranged in the form of Questions and Answers. Prepared especially for Students of Medicine, by Edward Martin, A. M., M. D. Second edition, revised and enlarged. Seventy-eight illustrations. Philadelphia: W. B. Saunders, 925 Walnut Street. 1893. Price, $1.00.

This little work of 166 pages forms No. 12 of Saunders' Question Compends.

Each paragraph begins with a question to which the paragraph is a comprehensive answer.

There are a number of books before the profession devoted to minor surgery, and several of them have been noticed in these pages. The present specimen is very good for the purpose intended. The subject of bandaging is extensively treated, as well as fracture dressings generally. Antiseptics, sponges, and antiseptic dressings are also noticed.

The volume has been before the profession for some time. The present issue is a thoroughly revised edition, and the illustrations have been redrawn and engraved, and an entirely new set of bandaging cuts inserted; for these, as well as the descriptions, the author has been indebted to the *American Text-Book of Surgery.*

